# Peptidoglycan *O*-acetylation increases in response to vancomycin treatment in vancomycin-resistant *Enterococcus faecalis*

**DOI:** 10.1038/srep46500

**Published:** 2017-04-13

**Authors:** James D. Chang, Erin E. Foster, Ashley G. Wallace, Sung Joon Kim

**Affiliations:** 1 Department of Chemistry and Biochemistry, Baylor University, Waco, Texas 76798, United States

## Abstract

Vancomycin resistance is conferred upon vancomycin-resistant enterococci (VRE) through the replacement of peptidoglycan (PG) stem terminal d-Ala-d-Ala with d-Ala-d-Lac. The d-Ala-d-Lac incorporation can affect both the fitness and virulence of VRE. Here we comprehensively investigate the changes to PG composition in vancomycin-resistant *Enterococcus faecalis* following the growth in presence of vancomycin using liquid chromatography-mass spectrometry. Using high-resolution mass spectrometry, 104 unique muropeptides fragments were identified and the relative abundance of each fragment was accurately quantified by integrating the ion current of a selected ion using extracted-ion chromatogram. The analysis indicates reduced PG cross-linking, increased carboxypeptidase activities, increased N-deacetylation, and increased *O*-acetylation in VRE when grown in the presence of vancomycin. We found that *O*-acetylation preferentially occurred on muropeptides fragments with reduced cross-linking with a pentapeptide stem that terminated in d-Ala-d-Lac. These findings show that *O*-acetylation preferentially occurred in regions of the cell wall with reduced PG cross-linking on PG units that have stems terminating in d-Ala-d-Lac, serving as markers to prevent both the PG-stem modification by carboxypeptidases and the cell wall degradation by autolysins. Accurate quantitative PG composition analysis provided compositional insights into altered cell wall biosynthesis and modification processes in VRE that contribute to lysozyme resistance and enhanced virulence for VRE grown in the presence of vancomycin.

The cell wall in Gram-positive bacteria, instead of being merely a static barrier, is a dynamic organelle that changes its structure and composition in response to the external stimuli^1^,^2^, including exposure to antibiotics^3^. An example of well-known cell wall modification is the mechanism of vancomycin resistance in vancomycin-resistant enterococci (VRE). Vancomycin is a glycopeptide antibiotic that binds to the d-Ala-d-Ala terminus of lipid II to inhibit the transglycosylation step of peptidoglycan (PG) biosynthesis, and it is considered one of the most effective therapeutic agents against serious infections by methicillin-resistant *Staphylococcus aureus* (MRSA). However, the emergence of VRE poses a serious threat to public health^4^ as the use of vancomycin against MRSA infections frequently leads to the selection of VRE as an alternative pathogen, with VRE as result becoming one of the leading nosocomial agents^5^. In VRE, high-level vancomycin resistance is conferred by either transposable elements *vanA* or *vanB*, where both encode for a number of proteins that alter the terminal d-Ala-d-Ala of PG stem to d-Ala-d-Lac (Fig. 1a)^6^,^7^. This depsipeptide substitution reduces vancomycin’s binding affinity to the PG-stem structure by a 1000-fold^8^, rendering vancomycin ineffective against VRE with minimal inhibition concentrations that often exceed 1024 μg/mL^9^.

Although the incorporation of d-Ala-d-Lac into cytoplasmic and membrane bound PG precursors Park’s nucleotide and lipid II is essential for vancomycin resistance, depsipeptide containing PG itself has never been identified from isolated cell walls of VRE. It is unknown how much of depsipeptide substituted PGs are incorporated into the cell wall and how they are modified following the induction of vancomycin resistance in VRE. These cell wall modifications are likely to play an essential role in bacterial fitness, persistence, virulence, and resistance to other antimicrobial agents. In this report we investigate the changes to PG composition of vancomycin-resistant *Enterococcus faecalis* (ATCC 51299), VRE of *vanB* type, using liquid chromatography-mass spectrometry (LC-MS) to provide insights into altered cell wall biosynthesis and PG modifications following the induction of vancomycin resistance.

## Results and Discussion

### Accurate Quantification of Muropeptides by LC-MS

Isolated cell walls of VRE or vancomycin-susceptible *E. faecalis* (VSE) are digested with mutanolysin, which is a *N*-acetylmuramidase that cleaves the β_1,4_ glycosidic bond between *N*-acetylmuramic acid (MurNAc) and *N*-acetylglucosamine (GlcNAc) of PG. Mutanolysin digestion of cell walls results in a complex mixture of muropeptide fragments not amenable to conventional spectral absorption measurements required for accurate quantification through traditional means. This difficulty arises from multiple muropeptide species that co-elute during the chromatographic separation, obfuscating identification and quantification of muropeptide peaks solely based on the absorption integral of the elutants. To circumvent this complexity, earlier studies have relied on treatment of cell walls with hydrofluoric acid to remove PG modifications prior to the LC-MS analysis. While this step significantly enhanced chromatographic resolution and separation required for the traditional analysis by reducing the variation in muropeptide species, rich muropeptide diversity that encoded for PG modifications were lost during the treatment.

**Figure 1:**
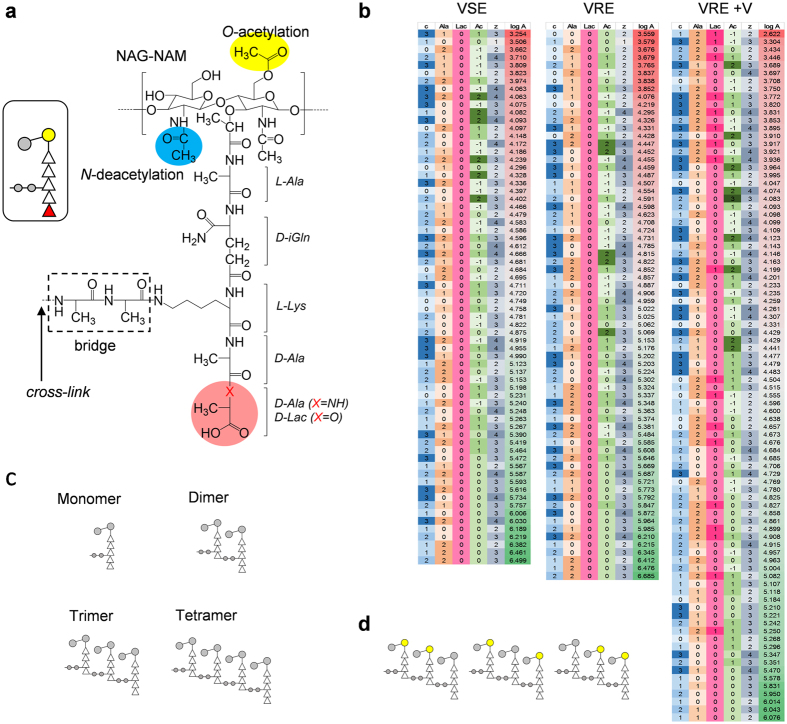
Structure of a peptidoglycan subunit and observed muropeptide fragments. (**a**) Chemical structure of a PG subunit is shown with modifications examined in this study highlighted. Disaccharide backbone and peptide stem are modified by O-acetylation of MurNAc (yellow), N-deacetylation of GlcNAc (blue), substitution and/or removal of terminal d-Ala-d-Ala (pink), and cross-linking to the adjacent PG stem (green). Schematic figure representing a subunit is shown in inset. Mutanolysin used to generates the fragments cleaves the GlcNAc-MurNAc β_1–4_ glycosidic linkage in PG. (**b**) Every identified muropeptide ion is described by its cross-linking (“c”), number of terminal d-Ala (“Ala”), depsipeptide substitution to d-Lac (“Lac”), acetylation state (“Ac”), charge state (“z”), and abundance (“logA”). The abundances are calculated by taking Log_10_ of averaged area under curve from extracted ion chromatograms (n = 3). Darker shades under ion descriptions indicate higher values, while abundances are colored green to red for increasing values. **c**) Muropeptide oligomers with increasing number of cross-links (0 to 3) are schematically depicted. (**d**) Figures of trimers with different possible combinations of O-acetylation that give arise to the acetylation number of +2 are shown.

Our approach differs by i) preserving the PG modifications by forgoing the acid treatment, ii) use of high resolution mass spectrometry (HRMS) for improved detection and identification of muropeptides, and iii) direct quantification of muropeptides by integrating extracted-ion chromatogram (XIC) of the selected ions. Detection and identification of such complex mixture is nonetheless still challenging and time consuming due to the possibility of each PG subunit undergoing multiple modifications (Fig. 1a)^10^,^11^. Hence the number of all possible chemical structures for muropeptides grows combinatorially with increase in the size of PG fragment (Fig. 1c). In order to facilitate the identification of complex muropeptide fragments, observed accurate mass by HRMS was screened against calculated muropeptide mass library for all possible combinatorial modifications generated *in silico* using MATLAB (MathWorks). From the library of 5328 possible ions, 104 unique muropeptide fragment ions were identified and selected for the quantitative analysis. Figure 1b shows the list of selected muropeptide fragments where each row represents multiple isomers that share a common PG modification. For example, a row is used to represent a pentapeptide-stem containing PG trimer with two *O*-acetylations. Based on the positions of *O*-acetylation (Fig. 1d), this sub group is comprised of 3 isomers.

For the accurate quantification of muropeptides, XICs of the selected ions were integrated and the relative abundance of each ion in respect to the sum of all selected ions calculated. The advantage of this approach is that even with multiple muropeptides co-eluting during the chromatographic separation, each muropeptide species is clearly resolved in the mass-charge dimension by XIC of unique m/z value. Although there may be differences in ionization efficiencies among muropeptide species, this was not considered critical as the analysis relied on the relative compositional changes associated with the induction of vancomycin resistance. Another key implementation crucial for the accurate quantification of 104 unique muropeptides was the integration of isotopic distribution for each selected ion, as the isotopic distribution varies significantly based on the chemical composition of muropeptides. Larger muropeptides with more dispersed isotopic distributions would otherwise have been underestimated, as XICs are derived from the abundance of a single peak from the distribution. The isotopic distribution and necessary correction of abundance for each identified ion were calculated using in-house MATLAB program utilizing the built-in mass spectrometry functions.

### PG Acetylation and Cross-linking

**Figure 2:**
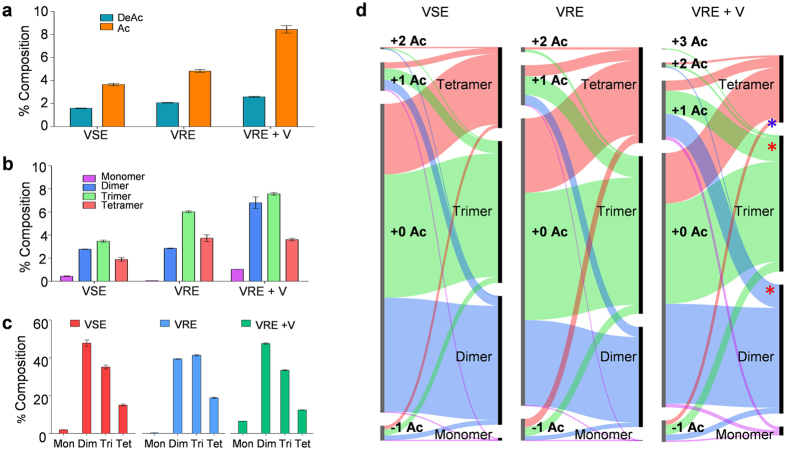
Acetylation state and crosslinking. (**a**) Schematic drawing corresponding to PG monomer, dimer, trimer, and tetramer without any modifications are shown. (**b**) Proportions of total PG subunits with both net positive (orange) and negative (blue) acetylation states increase when VRE is grown with vancomycin. (**c**) Proportion of subunits with at least one O-acetylation increases for all fragments in presence of vancomycin. (**d**) Breakdown of each acetylation state according to the acetylation state and cross-linking shows an increase in overall acetylation for all degrees of crosslinking. All error bars represent 95% confidence interval (n = 3).

One of more striking changes to the cell wall composition that occurs in VRE following the induction of vancomycin resistance is the increase in both PG *O*-acetylation and *N*-deacetylation (Fig. 2a). Acetylation states of −1, 0, +1, and +2 were considered for the analysis. For example, on a dimer acetylation state of −1 represents a dimer with one *N*-deacetylated GlcNAc, 0 represents unmodified PG dimer, +1 represents *O*-acetylation of one MurNac, and +2 for *O*-acetylation of both MurNAc. Each step-wise increment in acetylation state is accompanied with the mass increase of 42 Da. Sample mass spectra of PG dimers with acetylation states of 0 and +1, identified from VRE grown with vancomycin, are shown in supplementary Fig. S1. Mass spectrum of collision-induced dissociation product ions from a parent ion, +1 acetylated PG dimer with a tripeptide stem is shown in Fig. S2. Percent composition for all muropeptides that have one or higher acetylation state for VRE grown with vancomycin is 19.811% ± 2.363%. This is approximately 60% increase from acetylated muropeptides in VRE (12.361% ± 0.794%) and 120% increase from VSE grown without vancomycin (9.135% ± 0.941%). Increased PG *O*-acetylation and *N*-deacetylation^12^,^13^,^14^ represent significant modifications to VRE cell wall in response to vancomycin treatment.

Acetylated muropeptides were classified based on their number of cross-links and shown as a bar graph in Fig. 2b with monomers shown in purple, dimers in blue, trimers in green, and tetramers in orange. For VRE grown with vancomycin, while the monomers show the largest relative increase in *O*-acetylation with more than 16-fold increase (from 0.063% ± 0.014% to 1.036% ± 0.025%), *O*-acetylated dimers and trimers remain the most abundant species. Preferential *O*-acetylation of PG dimers and trimers are clearly noticeable, but the tetramers show a slight reduction in PG *O*-acetylation. These trends suggest that the cell wall modification through acetylation state change is neither random nor uniform, but is a selective process.

To further investigate the relationship between PG acetylation and cross-linking, PG cross-linking efficiency (*ρ*_*CL*_) was determined for VRE as a function of presence of vancomycin. The *ρ*_*CL*_ was calculated through summations of integrated ion current XICs from selected muropeptide species (Fig. 1b) by dividing the total number of cross-links present with the total number of PG subunits. *ρ*_*CL*_ for VRE grown with vancomycin (0.603 ± 0.002) shows 6% decrease in comparison to *ρ*_*CL*_ of VRE grown without vancomycin (0.641 ± 0.002), indicating a small reduction in PG cross-linking to the level comparable for VSE grown without vancomycin as measured by *ρ*_*CL*_ (0.620 ± 0.010). The effect of vancomycin resistance induction on VRE cell wall cross-linking is visible by altered oligomeric muropeptide profiles shown in Fig. 2c as increases in abundances of monomeric and dimeric muropeptide species and decrease in larger oligomers. Thus, the increase in PG *O*-acetylation is accompanied by reduced PG cross-linking for VRE grown in presence of vancomycin.

The correlation between PG acetylation and cross-linking is visualized by plotting simultaneously changes to the both on a flow-bar graph (Fig. 2d). The left axis consists of bar segments organized in an ascending order of acetylation states (Ac) from −1 to +3 with bar lengths proportional to the relative abundance as quantified through normalized summed integrals from XIC ion current of muropeptides for the corresponding acetylation state. The right axis shows bars with lengths proportional to the fraction of muropeptides with increasing cross-link. The width of interconnecting bars, which “flow” between two vertical axes, represents the fraction of muropeptides with shared modifications. While the flow-bar graph profiles of VRE and VSE grown in absence of vancomycin are similar, the profile of VRE grown in presence of vancomycin displays the following pronounced changes: i) large increase in *O*-acetylation of dimers and trimers (red stars), ii) decrease in *N*-deacetylation (−1) of tetramers (blue star), and iii) hyperacetylated trimers with the acetylation state of +3 where every MurNAc is *O*-acetylated. Preferential *O*-acetylation of dimers suggests that *O*-acetylation occurred in the region of cell wall with low PG cross-linking.

### PG Acetylation and Stem Length

**Figure 3:**
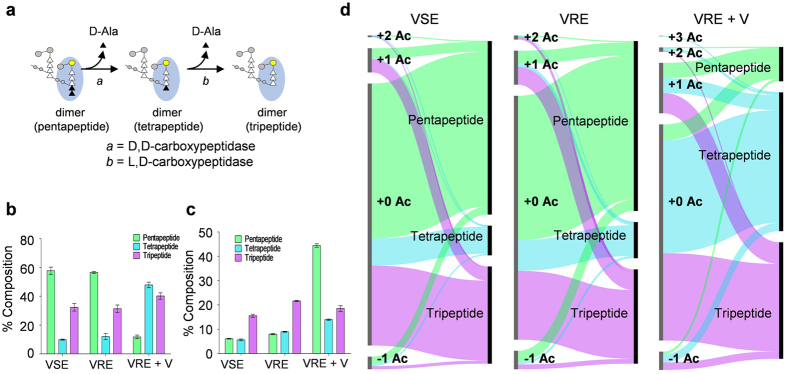
PG acetylation and peptide stem length. (**a**) Schematic drawing of monomer pentapeptide, tetrapeptide, and tripeptide and their associated stem length modifications are shown as reference. No modifications other than the PG stem terminal editing is shown. (**b**) Overall PG composition by peptide-stem length shows simultaneous increase in tetrapeptide and decrease in pentapeptide with addition of vancomycin. (**c**) Proportion of subunits with one or more O-acetylation markedly increase for pentapeptides in presence of vancomycin. (**d**) Breakdown of fragments by the acetylation state and peptide-stem length shows that upon addition of vancomycin, fragments of all peptide stem lengths are equally acetylated. +0 Ac fragments are composed of mostly penta- and tripeptides, but with addition of vancomycin tetra- and tripeptides become the majority. All error bars represent 95% confidence interval (n = 3).

Another major change to the PG composition observed in VRE grown with vancomycin is the PG-stem modification by l,d- and d,d-carboxypeptidases (Fig. 3a)^15^. The distribution of muropeptides with the cross-link acceptor-stem structure terminating with pentapeptide, tetrapeptide, and tripeptide is shown in Fig. 3b. For VRE grown with vancomycin, the majority of muropeptides are found with tetrapeptide-stem structure indicative of high d,d-carboxypeptidase activity, and accumulation of muropeptides with tripeptide stem eventually follows. The distribution of muropeptides with at least one *O*-acetylation as a function of PG-stem length is shown in Fig. 3c. Although the fraction of muropeptides containing pentapeptide stem decreases in VRE from increased carboxypeptidases activities (Fig. 3b), the abundance of *O*-acetylated pentapeptide-stem muropeptides increases by approximately 5 fold following the induction of vancomycin resistance (from 7.993% ± 0.694% to 44.441% ± 2.244%). These two trends imply that either *O*-acetylation prevented modification of PG stems by d,d-carboxypeptidases or the pentapeptide PG-stem motif is recognized as the site of *O*-acetylation.

To characterize the correlation between PG acetylation and stem modification, PG compositions as sorted by these two modifications are simultaneously visualized on flow-bar graphs (Fig. 3d). The left axis represents the fraction of muropeptides with increasing acetylation state, and the right axis muropeptides with increasing stem lengths. For VSE and VRE grown without vancomycin, PG with +1 acetylation state are composed from approximately equal proportions of pentapeptide- and tripeptide-stem muropeptides. With addition of vancomycin, the composition of +1 acetylation state PG for VRE changes with a significant increase in tetrapeptide-stem muropeptides to the point where muropeptides with tripeptide stem (36.033% ± 4.585%), tetrapeptide stem (33.793% ± 3.154%), and pentapeptide stem (30.174% ± 2.117%) all contribute in approximately equal proportions (Fig. 3d, right). Of particular note is that the proportion of +1 acetylated muropeptides with a pentapeptide stem in VRE does not change with the induction of vancomycin resistance, suggesting that *O*-acetylation of muropeptides with pentapeptide stem prevents PG-stem modification by d,d-carboxypeptidase. Furthermore, accumulation of *O*-acetylated muropeptides with tetrapeptide stem is consistent with *O*-acetylation also inhibiting l,d-carboxypeptidase activity in VRE.

### PG Cross-link and Stem Length

**Figure 4:**
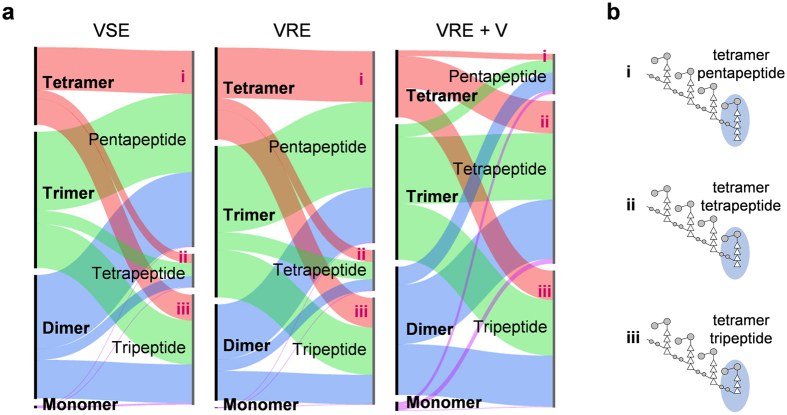
PG cross-linking and peptide-stem length. (**a**) Breakdown of fragments by cross-linking and peptide-stem length shows the distinct shift of peptide-stem population towards tetrapeptides with vancomycin addition. (**b**) Schematic drawing of tetramers with various lengths of peptide-stem highlights the fact that the final subunit of every oligomer is where the cross-linking occurs.

The correlation between PG cross-link and stem length in VRE is visualized using a flow-bar graph (Fig. 4a). The left axis consists of bars with lengths proportional to the fraction of muropeptides with increasing number of cross-links, and bars on the right axis represent fraction of muropeptides with increasing stem lengths, of which schematic representations of tetramers with (i) pentapeptide, (ii) tetrapeptide, and (iii) tripeptide PG-stem structure are shown as an example in Fig. 4b. The flow-bar graph makes it apparent that PG compositions of VSE and VRE grown in absence of vancomycin are similar to each other, with the majority of muropeptides having pentapeptide-stem structure, tripeptide- and tetrapeptide-stem muropeptide species subsequently following in abundance. This profile changes in VRE upon addition of vancomycin with the reduction in proportion of pentapeptide-stem structured muropeptides, which is consistent with high d,d-carboxypeptidase activity. Simultaneously, the proportion of tetrapeptide-stem muropeptides increases by more than five-fold, indicating the inhibition of l,d-carboxypeptidase activity. As muropeptides with tetrapeptide-stem structure in VRE are found to be preferentially *O*-acetylated (Fig. 3d), this also implies that l,d-carboxypeptidase activity was inhibited by the *O*-acetylation.

### PG Acetylation and Depsipeptide Substitution

**Figure 5:**
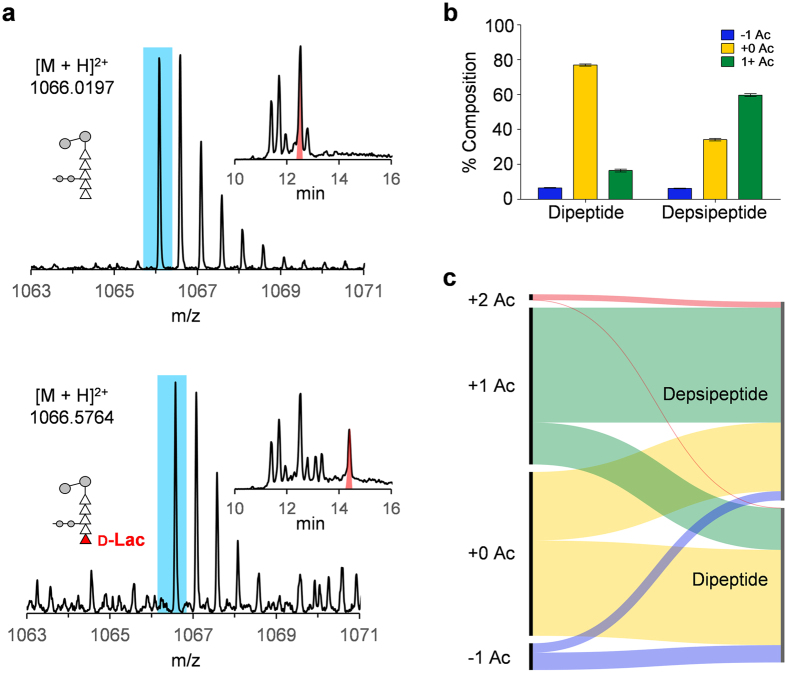
PG acetylation and depsipeptide substitution. (**a**) Mass spectra and extracted ion chromatograms (XIC) for the unmodified dimer pentapeptide and its d-Ala-d-Lac substituted counterpart are shown. The substitution manifests itself as mass increase of 1 Da, or ½ m/z value for doubly charged ion (blue dotted line), and with different retention time (red). (**b**) Composition of PG pentapeptides shifts towards +1 or higher acetylation state in VRE upon the induction of vancomycin resistance. (**c**) Breakdown of each acetylation state according to the PG stem terminal displays a preference for depsipeptide to have one or more acetylated subunits. All error bars represent 95% confidence interval (n = 3).

The incorporation of depsipeptides into PG-stem structure is the hallmark of vancomycin resistance in VRE. Muropeptides with a depsipeptide stem exhibit lengthened chromatographic retention time and an increase of 1 mass unit compared to the dipeptide containing muropeptides (Fig. 5a). Quantitative analysis has determined that 57.328% ± 1.840% of muropeptides with a pentapeptide stem from VRE grown with vancomycin have d-Ala-d-Lac substitution. Furthermore, more than half of muropeptides with a d-Ala-d-Lac substituted stem (57.584% ± 2.827%) have one or more *O*-acetylation (Fig. 5b). In contrast, a large fraction of muropeptides with d-Ala-d-Ala stem are found without any acetylation (77.907% ± 2.133%). The relative ratio of muropeptides in +0 acetylation state with dipeptide to depsipeptide is 1.0:0.8, but for +1 acetylation state the ratio increases to 1.0:3.4. Therefore, muropeptides with the d-Ala-d-Lac stem structure are preferentially *O*-acetylated, and this preference is clearly visible in the flow-bar diagram as shown in Fig. 5c. The majority of d-Ala-d-Lac containing muropeptides have +1 and 2 acetylation state, but for muropeptides containing d-Ala-d-Ala stem structure +0 acetylation state composes the majority. Interestingly, *N-*deacetylated (−1 Ac) muropeptides display the opposite trend by primarily having d-Ala-d-Ala stem structure.

PG *O*-acetylation has been shown to inhibit the autolysin activity^16^. Thus preferential *O*-acetylation of muropeptides with d-Ala-d-Lac stem (Fig. 5c) suggests that the newly synthesized nascent PG in VRE containing d-Ala-d-Lac stem is marked with *O*-acetylation to prevent autolysin degradation and PG-stem modification by carboxypeptidases (Fig. 5b). In contrast, the old cell wall containing d-Ala-d-Ala stem structure is preferentially *N*-deacetylated. Since both *O*-acetylation and *N*-deacetylation of PG contribute to increased lysozyme resistance, we speculate that these cell wall modifications can potentially contribute to evasion of host’s innate immune response^16^,^17^. Therefore, vancomycin therapy against VRE may indirectly cause increased virulence and transmittance in both the patient undergoing antibiotic therapy and the healthcare setting where the therapy is being administered.

## Materials and Methods

### Cell Wall Isolation and Digestion for LC-MS

Overnight cultures of VRE (ATCC 51299) and VSE (ATCC 29212) grown in brain-heart infusion media at 37 °C with 180 RPM orbital shaking were used to inoculate flasks containing 100 mL of tryptic soy broth (TSB) (1% v/v). Vancomycin resistance was induced by addition of vancomycin at the time of inoculation (6 μg/mL). Bacteria were harvested at stationary phase (OD_600_ > 0.8) by centrifugation at 4 °C at 4750 RPM (Allegra X-15R with SX4750 rotor, Beckman Coulter) for 12 min. Pellets were resuspended in phosphate buffered saline (PBS) and sterilized by immersing in a boiling water bath for 30 min. Samples were bead beat (Disruptor Genie, Scientific Industries) with 0.5 mm diameter glass beads for 8 one-min cycles with 1 min of rest in between agitation. Beads and other contaminants were removed using Steriflip 20 μm nylon vacuum filter (EMD Millipore). Crude cell wall pellets were resuspended in 2 mL PBS, to which 8 mL of 2% sodium dodecyl sulfate (SDS) solution was added, then placed in a boiling water bath for 30 min. Boiled cell wall pellets had SDS removed from them by dividing the pellets into microcentrifuge tubes and washing with five 1 mL deionized water through centrifiguation. Isolated crude cell walls were resuspended in 2 mL of 50 mM Tris pH 8.0 buffer. DNase (200 μg) was added to the cell wall suspension and it was incubated at 37 °C for 24 hr at 80 rpm, which was followed by addition of trypsin (200 μg) for additional 24 hr of incubation. Cell walls were washed once and resuspended in 1 mL of Tris buffer.

To generate PG fragments for the LC-MS compositional analysis, 0.66 KU of mutanolysin (Sigma-Aldrich) to hydrolyze β_1,4_ glycosidic bonds in PG was added to the cell wall suspension at room temperature and the sample incubated for 24 hr. Additional 0.66 KU of mutanolysin was added to the mixture after the initial period, and it was further incubated for 24 hr, then the sample frozen and lyophilized (Labconco). Lyophilized mutanolysin-digested cell walls were dissolved in 1 mL of 0.375 M sodium borate buffer (pH 9.0) prepared with HPLC-grade water, and samples were reduced by addition of 10 mg of sodium borohydride (Fisher Scientific) in 960 μL borate buffer at room temperature for 30 min. The reduction was quenched by addition of 125 μL of 85% phosphoric acid. Reduced samples were frozen at −80 °C, and lyophilized. Prior to LC-MS analysis, lyophilized samples were resuspended in 1 mL of sample preparation buffer (1% trifluroacetic acid), centrifuge filtered, and cleaned up for LC-MS using 100 μL Pierce C18 tips (Thermo Scientific).

### Liquid Chromatography-Mass Spectrometry

Mutanolysin-digested muropeptide fragments were chromatographically separated using NanoACQUITY Ultra Performance Liquid Chromatography System (Waters). Reverse phase BEH C18 column (length of 100 mm and diameter of 75 μm) had bead size of 1.7 μm and pore size of 130 Å. Chromatographic separation of mutanolysin-digested PG was carried out by injecting 1 μL of the sample from a 5 μL sample loop to the column under isocratic condition of 99% buffer A (99.8% anhydrous methanol with 0.1% formic acid) and 1% buffer B (100% acetonitrile) for 5 min, then a linear gradient to 50% buffer B was applied for 30 min for the separation. The column was regenerated under isocratic condition with 85% buffer B for 5 min, a linear gradient to 98% buffer A for 1 min, then isocratic at 98% buffer A for 23 min. The flow rate was kept constant (0.6 μL/min) throughout the analysis.

The sample was ionized by nanoflow electrospray ionization (ESI) with spray voltage of 35 V and capillary voltage of 3.5 kV. Synapt G2 High Definition Mass Spectrometer (HDMS) with Time-of-Flight (TOF) mass analyzer (Waters) was run in positive ion mode. Fibrinopeptide B (Glu-Fib) was used as an internal standard to correct for drift of the instrument. Data were analyzed using MassLynx (Waters) and MATLAB (MathWorks)^18^,^19^,^20^,^21^.

## Additional Information

**How to cite this article**: Chang, J. D. *et al*. Peptidoglycan *O*-acetylation increases in response to vancomycin treatment in vancomycin-resistant *Enterococcus faecalis. Sci. Rep.* **7**, 46500; doi: 10.1038/srep46500 (2017).

**Publisher's note:** Springer Nature remains neutral with regard to jurisdictional claims in published maps and institutional affiliations.

## Supplementary Material

Supplementary Information
