# Supplementary material for: Peptidoglycan O-acetylation increases in response to vancomycin treatment in vancomycin-resistant Enterococcus faecalis
Source: Sci Rep. 2017 Apr 13;7:46500. doi: 10.1038/srep46500 (PMC5390252; doi:10.1038/srep46500)
Supplement: Supplementary Information [file srep46500-s1.pdf]

Peptidoglycan *O*-acetylation increases in response to vancomycin treatment in vancomycin-resistant *Enterococcus faecalis*

James D. Chang[1], Erin E. Foster[1], Ashley G. Wallace[1], Sung Joon Kim[1,2]

[1]Department of Chemistry and Biochemistry, Baylor University, Waco, Texas 76798, United States.

[2]To whom the correspondence should be addressed: Tel: 1-254-710-2531; e-mail address: Sung_J_Kim@baylor.edu; Fax: 1-254-710-4272.

Supplementary Figures

Fig. S1.

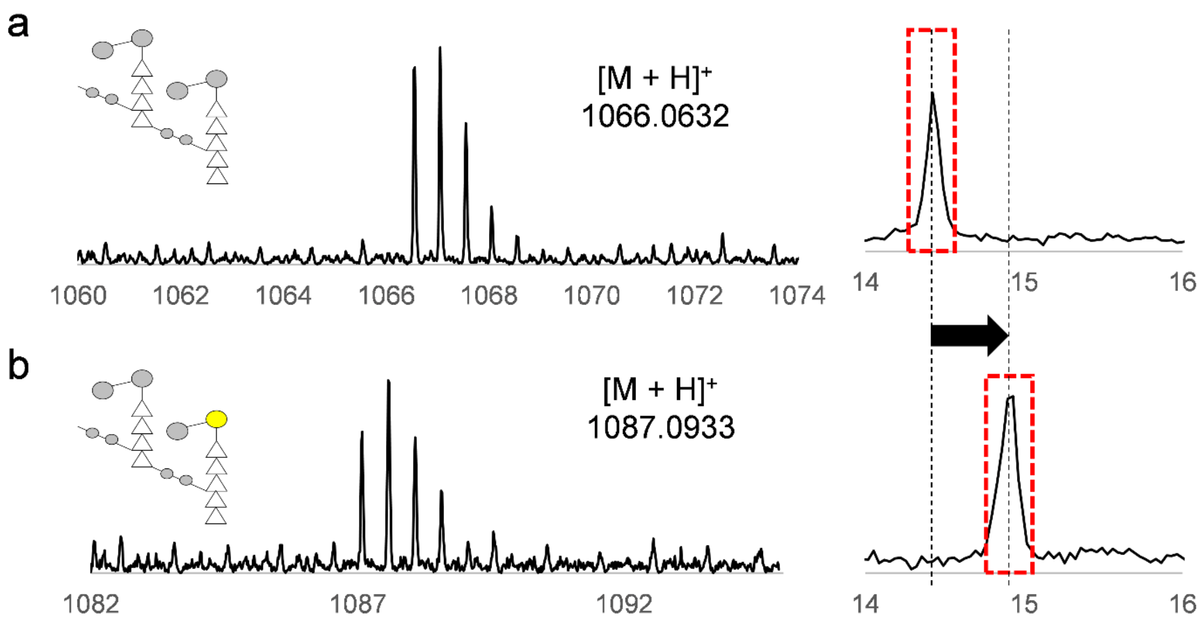

Supplementary Figure 1. Example spectra and XIC of identified fragments. a) Schematic drawing of the muropeptide fragment ion, mass spectrum, and XIC for dimer pentapeptide at 0 Ac with +2 charge are shown. **b)** Singly O-acetylated counterpart of the same muropeptide has different m/z value and retention time as indicated by the red box in XICs. O-acetylation is denoted by a yellow circle.

Fig. S2.

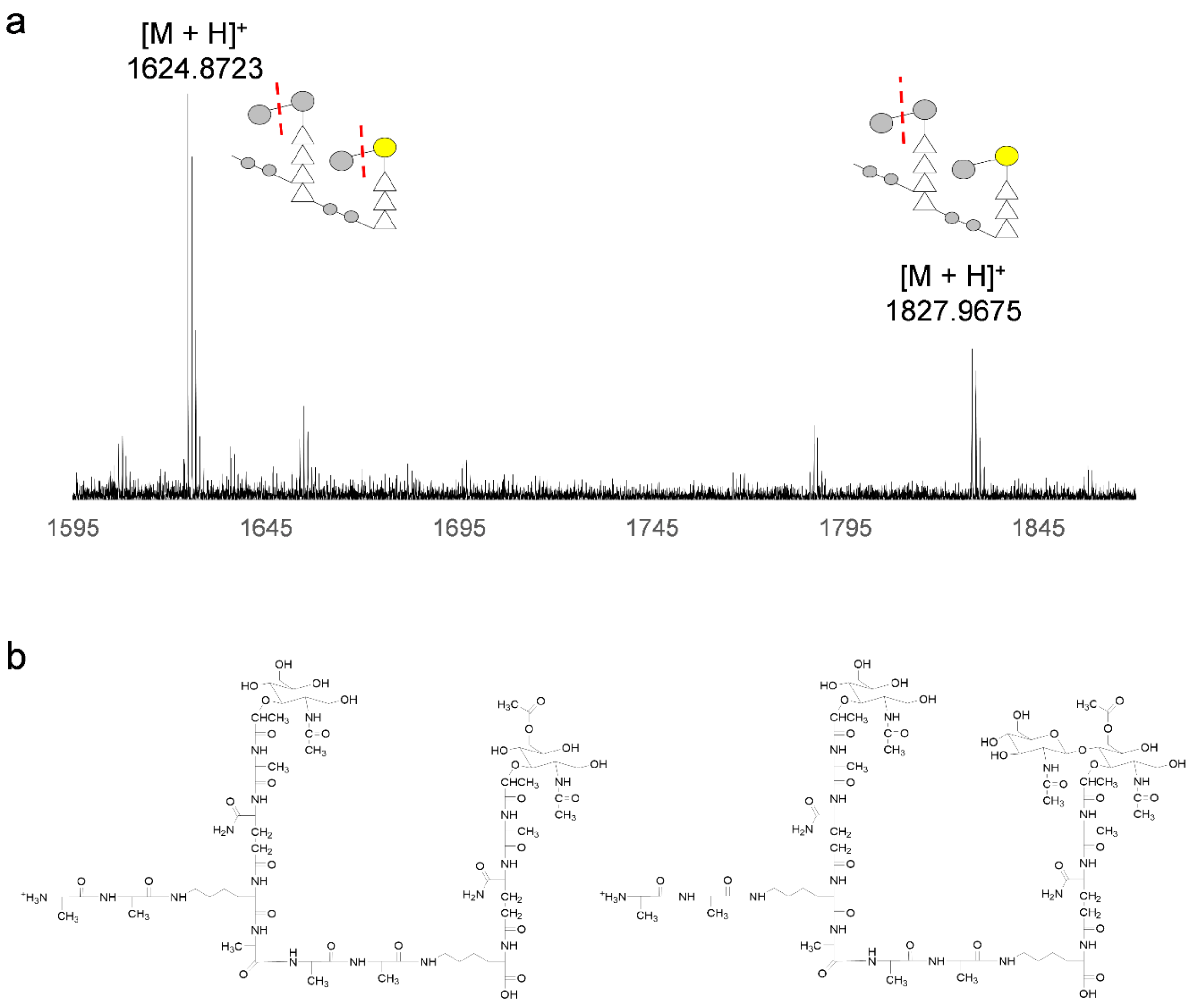

Supplementary Figure 2. MS/MS spectrum of a sample peptidoglycan. a) MS/MS spectrum from a dimer pentapeptide muropeptide with +1 acetylation state shows the CID product ions with two and one GlcNAc's fragmented away. Red lines indicate the location of CID fragmentation. m/z values shown above the peaks are for (b+) ion products of fragmentation. **b)** Chemical structures for product ions of dimer pentapeptide at +1 acetylation state with two and one GlcNAc fragmented away.
